# Lower airway microbiota compositions differ between influenza, COVID-19 and bacteria-related acute respiratory distress syndromes

**DOI:** 10.1186/s13054-024-04922-2

**Published:** 2024-04-22

**Authors:** Sébastien Imbert, Mathilde Revers, Raphaël Enaud, Arthur Orieux, Adrian Camino, Alexandre Massri, Laurent Villeneuve, Cédric Carrié, Laurent Petit, Alexandre Boyer, Patrick Berger, Didier Gruson, Laurence Delhaes, Renaud Prével

**Affiliations:** 1 HU Bordeaux, Mycology-Parasitology Department, CIC 1401, 33000 Bordeaux, France; 2 Centre de Recherche Cardio-Thoracique de Bordeaux, Inserm UMR 1045, Univ Bordeaux, 33000 Bordeaux, France; 3 CHU Bordeaux, CRCM Pédiatrique, CIC 1401, 33000 Bordeaux, France; 4 CHU Bordeaux, Medical Intensive Care Unit, 33000 Bordeaux, France; 5 CH Pau, Intensive Care Unit, 64000 Pau, France; 6 CH Pau, Microbiology Laboratory, 64000 Pau, France; 7 d CHU Bordeaux, Surgical Intensive Care Unit, 33000 Bordeaux, France; 8 Medical Intensive Care Unit, Pellegrin Hospital, Place Amélie Raba-Léon, 33076 Bordeaux, France

**Keywords:** Acute respiratory distress syndrome, Mycobiota, Microbiota

## Abstract

**Background:**

Acute respiratory distress syndrome (ARDS) is responsible for 400,000 deaths annually worldwide. Few improvements have been made despite five decades of research, partially because ARDS is a highly heterogeneous syndrome including various types of aetiologies. Lower airway microbiota is involved in chronic inflammatory diseases and recent data suggest that it could also play a role in ARDS. Nevertheless, whether the lower airway microbiota composition varies between the aetiologies of ARDS remain unknown. The aim of this study is to compare lower airway microbiota composition between ARDS aetiologies, i.e. pulmonary ARDS due to influenza, SARS-CoV-2 or bacterial infection.

**Methods:**

Consecutive ARDS patients according to Berlin’s classification requiring invasive ventilation with PCR-confirmed influenza or SARS-CoV-2 infections and bacterial infections (> 105 CFU/mL on endotracheal aspirate) were included. Endotracheal aspirate was collected at admission, V3-V4 and ITS2 regions amplified by PCR, deep-sequencing performed on MiSeq sequencer (Illumina®) and data analysed using DADA2 pipeline.

**Results:**

Fifty-three patients were included, 24 COVID-19, 18 influenza, and 11 bacterial CAP-related ARDS. The lower airway bacteriobiota and mycobiota compositions (β-diversity) were dissimilar between the three groups (*p* = 0.05 and *p* = 0.01, respectively). The bacterial α-diversity was significantly lower in the bacterial CAP-related ARDS group compared to the COVID-19 ARDS group (*p* = 0.04). In contrast, influenza-related ARDS patients had higher lung mycobiota α-diversity than the COVID-19-related ARDS (*p* = 0 < 01).

**Conclusion:**

Composition of lower airway microbiota (both microbiota and mycobiota) differs between influenza, COVID-19 and bacterial CAP-related ARDS. Future studies investigating the role of lung microbiota in ARDS pathophysiology should take aetiology into account.

To the Editor,

Acute respiratory distress syndrome (ARDS) is still responsible for about 400,000 deaths per year worldwide with only few improvements despite five decades of intensive research [1, 2]. Failure in the development of new therapeutics could be explained by the fact that ARDS is a highly heterogeneous syndrome including both various types of aetiologies and extremely clinically and biologically different patients [3]. One approach suggested to provide insights in ARDS care is defining subphenotypes according to biological, radiological or causal classification [3]. Lungs have long been thought to be a sterile environment but recent development of culture-independent genomics methods revealed the existence of a lower airways microbiota, established within days to weeks after birth, which is crucial for immune system maturation and maintenance [4]. Lower airway microbiota is involved in numerous chronic inflammatory diseases and recent data suggest that it could also play a role in ARDS [5, 6]. Nevertheless, whether the lower airway microbiota composition varies between the aetiologies of ARDS remain unknown. The aim of this study is to compare lower airway microbiota composition (both bacterial and fungal kingdoms) between specific ARDS subphenotypes, i.e. pulmonary ARDS due to influenza, SARS-CoV-2 or bacterial CAP infections.

To this aim, every consecutive patient admitted to intensive care unit (ICU) in Pau and Bordeaux hospitals during the 2018–2019 season for community-acquired influenza-related and bacterial CAP-related ARDS patients was assessed for inclusion. Due to COVID-19 pandemic, inclusion of these patients stopped in 2020 and community-acquired coronavirus disease 2019 (COVID-19)-related ARDS patients were assessed for inclusion in March 2020 in Bordeaux hospital. ARDS was defined according to Berlin definition [7]. Influenza and SARS-CoV-2 infections were confirmed by polymerase chain reaction (PCR) (Argene®, Biomerieux), bacterial infection when > 10^5^ colony forming unit/mL bacteria were isolated on endotracheal aspirate. Patients were included if endotracheal aspirate was performed within 24 h after admission to ICU. Data were prospectively recorded by physicians in charge of the patient. Microbiota analysis were performed as previously described [8, 9]. Briefly, desoxyribonucleic acid (DNA) extraction from endotracheal aspirate was performed using QIAamp® PowerFaecal® Pro DNA kit (QIAgen®, Valencia, CA, USA) after mechanical lysis. V4 regions of the bacterial 16S rRNA encoding gene and the internal transcribed spacer 2 (ITS2) of the fungal rDNA regions were amplified by PCR. The respective primers used to amplify these loci were as follows: 16S-forward, TACGGRAGGCAGCAG; 16S-reverse, CTACCNGGGTATCTAAT; ITS2-forward, GTGARTCATCGAATCTTT; and ITS2-reverse, GATATGCTTAAGTTCAGCGGGT. After deep sequencing (2 × 250 bp paired-end, MiSeq sequencer, Illumina®, San Diego, CA, USA), raw data were processed through the DADA2 pipeline. Mock communities and negative controls (three from the DNA extraction step with unloaded swabs and three from the PCR amplification step) were included. DNA extraction, PCR amplification step and sequencing were performed simultaneously for all samples. Bacterial and fungal α-diversity metrics (Simpson and Shannon indices) were generated using the phyloseq R package. For cross-sectional analyses, significant differences in α-diversity were determined using the Kruskall-Wallis test. Differences in beta-diversity between groups (measured using Bray Curtis dissimilarity) were tested using a permutational multivariate analysis of variance (PERMANOVA) from vegan R package with 10,000 permutations, while accounting for individual identity as a covariate. DeSeq2, ANCOM-BC and LEfSe analysis was performed from microbiomeMarker package. Statistical analysis was performed with the R studio program (version 1.3.1056 for Windows™); correction for multiple-testing was performed using the Benjamini–Hochberg false discovery rate (FDR) procedure, a p-value or FDR adjusted *p*-value equal to or less than 0.05 was considered statistically significant. Quantitative variables are presented as median and interquartile range (IQR) and compared by use of the Kruskall–Wallis test. Categorical variables are expressed as number of patients (percentage) and compared by mean of Fisher’s exact test. All statistical tests were 2-tailed and statistical significance was defined as *p* < 0.05.

Sixty-five patients were assessed for inclusion and 53 (82%) were included: 24 (45%) COVID-19, 18 (34%) influenza- and 11 (21%) bacterial CAP-related ARDS. Reason for non-inclusion were lack of AT performed before antimicrobial therapy initiation (3 COVID-19-, 2 influenza- and 1 bacterial CAP-related ARDS patients) and decline to participate (2 COVID-19-, 2 influenza- and 2 bacterial CAP-related ARDS patients). Every patient in the bacterial CAP-related ARDS had negative influenza A/B, SARS-COV-2, parainfluenza, rhinovirus, respiratory syncytial virus and metapneumovirus (Argene®, Biomerieux) PCR. Patients’ characteristics are reported in Table 1. Isolated bacteria species were *Streptococcus pneumoniae* (3/11, 27%), *Escherichia coli* (3/11, 27%), *Klebsiella pneumoniae* (2/11, 18%), *Klebsiella aerogenes* (2/11, 18%) and methicillin-susceptible *Staphylococcus aureus* (1/11, 09%). The lower airway microbiota composition at admission varied according to the ARDS aetiology. First, both lower airways bacteriobiota and mycobiota were dissimilar according to the ARDS aetiology (*p* = 0.05 and *p* = 0.01 PERMANOVA, respectively) (Fig. 1A, B). Moreover, lower airway bacteriobiota α-diversity measured by the Simpson index was significantly lower in the bacterial CAP-related ARDS group compared to the COVID-19 ARDS group (median: 0.5, interquartile range (IQR) [0–0.7] *vs* 0.8 [0.7–0.9], *p* = 0.04, respectively), but not compared to the influenza-related ARDS group (median: 0.7, IQR [0–0.9], *p* = 0.46) (Fig. 1C). In contrast, the fungal α-diversity was significantly higher in the influenza-related ARDS compared to the COVID-19-related ARDS both compared with the Simpson index (median: 0.8, IQR [0.6–0.8] vs 0.7, IQR [0.5–0.8], *p* < 0.01, respectively), but not compared to the bacterial CAP-related ARDS group (median: 0.7, IQR [0.7–0.8], *p* = 0.13) (Fig. 1D). We obtained consistent results after exclusion of COPD patients from the analysis, except that dissimilarity of the lower airway bacteriobiota between the 3 groups did not reach significance (*p* = 0.15). Regarding bacteriobiota signatures, (i) in COVID-19-related ARDS patients, *Prevotella sp.* and *Streptococcus sp.* abundances were higher compared to influenza-related and bacterial CAP-related ARDS patients but *Bacteroides sp.* abundance only when compared to influenza-related ARDS patients, (ii) in bacterial CAP-related ARDS patients, *Acinetobacter sp.* and *Actinomyces sp.* abundances were higher compared to influenza-related ARDS patients and *Prevotella sp.* and *Pyramidobacter sp.* abundances were higher when compared to COVID-19-related ARDS patients, (iii) in influenza-related ARDS patients*, Corynebacterium sp.* abundances were higher compared to COVID-19-related ARDS patients and *Rothia sp.* and *Porphyromonas sp.* abundances.

**Table 1:** Characteristics of patients

	COVID-19 ARDS n = 24	Influenza ARDS n = 18	Bacterial CAP ARDS n = 11	*p*-value
Age (years)	57.5 [50.75;67]	58 [51.75;72.75]	66 [57;71.5]	0.38
Male sex	17/24 (71%)	10/18 (56%)	8/11 (73%)	0.56
**Chronic pulmonary disease**	**3/24 (12%)**	**9/18 (50%)**	**6/11 (55%)**	** < 0.01**
COPD	3/24 (12%)	5/18 (28%)	4/11 (36%)	0.2
**Asthma**	**0/24 (0%)**	**4/18 (22%)**	**1/11 (9.1%)**	**0.03**
Current smoking	4/24 (17%)	4/18 (22%)	3/11 (27%)	0.75
Chronic kidney disease	0/16 (0%)	1/18 (5.6%)	0/11 (0%)	1
Obesity	5/23 (22%)	5/18 (28%)	1/11 (9.1)	1
Diabetes mellitus	4/23 (17%)	3/18 (17%)	1/11 (9.1%)	1
Heart coronary disease	6/24 (25%)	4/18 (22%)	6/11 (55%)	0.18
High blood pressure	7/16 (44%)	6/18 (33%)	4/11 (36%)	0.92
Immunodepression	4/24 (17%)	0/18 (0%)	2/11 (18%)	0.11
Long-term systemic corticosteroids	1/24 (4.2%)	1/18 (5.6%)	0/11 (0%)	1
Antibiotics before admission	5/16 (31%)	3/10 (30%)	1/10 (10%)	0.5
Antifungals before admission	0/16 (0%)	0/10 (0%)	0/10 (0%)	1
PPI	2/24 (8.3%)	0/10 (0%)	1/11 (9.1%)	1
Metformin	1/23 (4.3%)	1/17 (5.9%)	1/11 (9.1%)	1
SAPS 2	47.5 [35.5;56.75]	45 [31.25;57.5]	68.5 [53.5;86]	0.08
Heart rate (/min)	91 [80;108]	98.5 [73;110.75]	90 [80;116]	0.85
Respiratory rate (/min)	26 [25.25;29.75]	25.5 [22.25;28]	32.5 [24.75;40]	0.22
Mean blood pressure (mmHg)	85 [78;97]	86.5 [78.5;101]	84 [78.75;93.25]	0.92
PaO2/FiO2	140 [132;204]	150 [121.7;206.2]	120 [91;144.5]	0.19
Neutrophils count (/mm^3^)	8340 [5227;11160]	4980 [3412;7750]	7460 [2465;10935]	0.07
Lymphocytes count (/mm^3^)	660 [540;862.5]	750 [402.5;1142.5]	340 [225;1070]	0.6
Fibrinogen (g/L)	9.6 [8.55;9.9]	6.5 [6.125;8.25]	7.7 [5.2;9.6]	0.06
CRP (mg/L)	189 [116;286]	47 [41;1,006]	159 [101;214]	0.15
**Septic shock**	**11/24 (46%)**	**3/18 (17%)**	**7/11 (64%)**	**0.026**
Acute kidney injury	6/16 (38%)	5/18 (28%)	6/11 (55%)	0.37
Day 28 survival	19/24 (79%)	15/18 (83%)	7/11 (64%)	0.48

Bold lines represent variables with statistical significant differences (*p*-value < 0.05)

Results are presented as number and percentages () for categorical variables and median and interquartile range [] for continuous variables. Categorical and continuous variables are compared by Fisher’s exact and Kruskal–Wallis tests, respectively. Bold lines represent variables with statistical significant differences (*p*-value < 0.05). Before admission refers to admission to intensive care unit. CAP: community-acquired pneumonia. COPD: chronic obstructive pulmonary disease. CRP: C-reactive protein. PPI: pump proton inhibitor. SAPS2: simplified acute physiology score 2

**Fig. 1:**
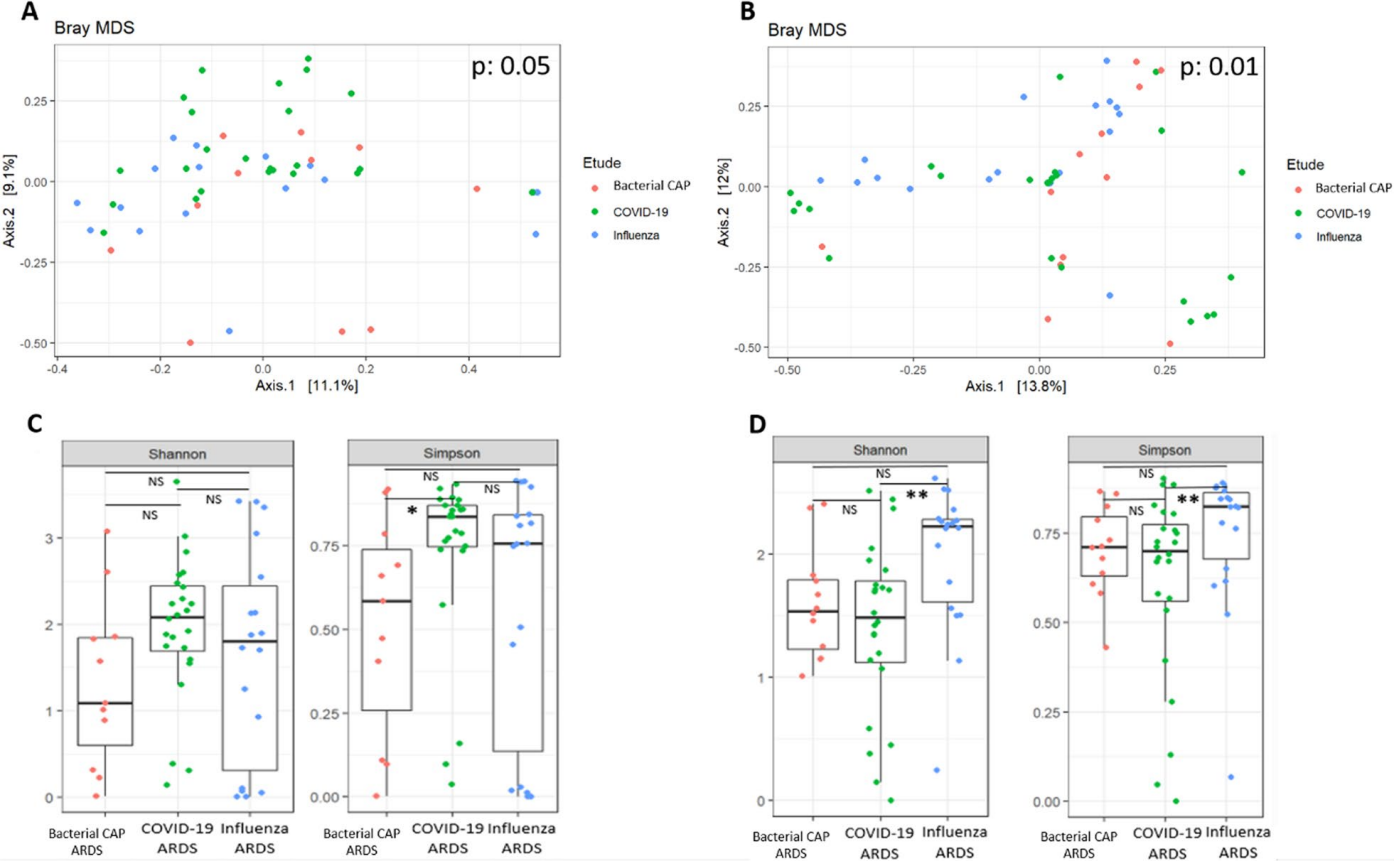
Lower airway microbiota compositions differ between influenza, COVID-19 and bacterial CAP-related acute respiratory distress syndromes. **A** Metric Bray–curtis analysis of β-diversity of lower airway bacteriobiota. **B** Metric Bray–curtis analysis of β-diversity of lower airway mycobiota. **C** Boxplot of estimated lower airway bacteriobiota α-diversity by Shannon and Simpson indices. **D** Boxplot of estimated lower airway mycobiota α-diversity by Shannon and Simpson indices. NS: non-significant difference, *p < 0.05, ** *p* < 0.01. The median reads counts were 7260 (interquartile range [IQR]: 419; 16,352) for 438 Amplicon Sequence Variants (ASVs) and the median fungal reads count was 171 (IQR: 93; 773) for 561 ASVs,

To the best of our knowledge, this study is the first to compare the lower airway microbiota composition between specific ARDS aetiologies. Influenza patient exhibited significant differences regarding the lower airway mycobiota composition which could also be influenced by the increased proportion of patients with chronic obstructive pulmonary disease in this group, a condition known to be associated with lower airway mycobiota perturbation [8, 10]. In ARDS, a relationship between the presence of gut-associated bacteria and mixed oral commensals within the lungs with lung inflammation has been demonstrated [5]. Nevertheless, highly heterogeneous patients with both pulmonary and extra-pulmonary ARDS were included and at a later course of disease (3–7 days after admission). Lower airways enrichment with *Pseudomonadaceae* but also with *Staphylococcus* was associated with increased lower airway inflammation, longer time to weaning from invasive mechanical ventilation and worse 30-days mortality [6]. Focusing on critically ill COVID-19 patients, lower airway enrichment with *Mycoplasma salivarium* (an oral commensal) was associated with worse outcomes [11]. Lower airway mycobiota has been poorly investigated, even more in ARDS. The potential role of lower airway mycobiota composition in influenza-related ARDS patients for the subsequent development of fungal superinfections needs to be further deciphered. Nevertheless, it is unlikely that it is the sole explanation as inter-kingdom crosstalk occurs within the lower airway microbiota [12], for instance *Pseudomonas aeruginosa* promoting the growth of *Aspergillus fumigatus* [13, 14]. The intensity of the compensatory local immunosuppressive response is also thought to promote the development of fungal superinfections [15]. These data suggest that lower airway microbiota composition could play a role in the development of subsequent superinfections and in the immune dysregulation involved in acute lung injury occurring during ARDS.

In fact, lower airway microbiota composition has been demonstrated to be associated with lower airway immune tone [16, 17], causality between immune imprinting and the microbial composition being confirmed by a murine study [18]. Yet, if lower airway microbiota can lead to increased inflammation, it can also be associated with immune exhaustion. This Janus effect of lower airway microbiota is of major importance when assessing the role of lung lower airway microbiota in ARDS as this microbiota vary during invasive mechanical ventilation [19] and can so have different immunomodulatory effects over time. Lower airway microbiota could so play a role in both early lung inflammation leading to lung injury at the acute phase of ARDS, and to immune exhaustion paving the way of subsequent ventilator-associated pneumonia later in the course of the disease.

Our study has some limitations. First, it includes a limited number of patients and needs further confirmation in larger cohorts. Second, it only concerns pulmonary ARDS and thus our data cannot be extrapolated to extra-pulmonary ARDS. But, this limit also increases internal validity as we have included highly homogeneous patients within the different groups. Third, patients with influenza-related ARDS had a higher proportion of COPD which is known to alter the composition of lung microbiota compared to healthy controls. Moreover, the impact of previous medications cannot be formerly excluded. Last, we do not have concomitant immune lung response to correlate lower airway microbiota with local inflammatory state. Hold together, these data suggest that both lower airway bacteriobiota and mycobiota compositions differ between pulmonary ARDS subgroups, *i.e.* influenza-, COVID-19 and bacterial CAP-related ARDS. Future studies investigating the role of lower airway microbiota in ARDS pathophysiology are warranted and should take aetiology into account.

## Data Availability

The 16S rRNA gene and ITS2 sequences have been submitted to the European Nucleotide Archive (Accession N° ERP134910). The scripts used for bioinformatics analysis during the current study are available in the Supplemental Materials of a previous study from our team (https://journals.asm.org/doi/10.1128/spectrum.05062-22).

## Abbreviations

ARDS: Acute respiratory distress syndrome
ASV: Amplicon sequence variant
COVID-19: Coronavirus disease 2019
DNA: Desoxyribonucleic acid
ICU: Intensive care unit
ITS2: Internal transcribed spacer 2
PERMANOVA: Permutational multivariate analysis of variance
PCR: Polymerase chain reaction
RNA: Ribonucleic acid
